# High-Responsivity Multilayer MoSe_2_ Phototransistors with Fast Response Time

**DOI:** 10.1038/s41598-018-29942-1

**Published:** 2018-08-01

**Authors:** Hyejoo Lee, Jongtae Ahn, Seongil Im, Jiyoung Kim, Woong Choi

**Affiliations:** 1 School of Materials Science & Engineering, Kookmin University, Seoul, 02707 South Korea; 2 Institute of Physics and Applied Physics, Yonsei University, Seoul, 03722 South Korea; 3 Department of Materials Science & Engineering, University of Texas at Dallas, Richardson, Texas 75080 USA

## Abstract

There is a great interest in phototransistors based on transition metal dichalcogenides because of their interesting optoelectronic properties. However, most emphasis has been put on MoS_2_ and little attention has been given to MoSe_2_, which has higher optical absorbance. Here, we present a compelling case for multilayer MoSe_2_ phototransistors fabricated in a bottom-gate thin-film transistor configuration on SiO_2_/Si substrates. Under 650-nm-laser, our MoSe_2_ phototransistor exhibited the best performance among MoSe_2_ phototransistors in literature, including the highest responsivity (1.4 × 10^5^ AW^−1^), the highest specific detectivity (5.5 × 10^13^ jones), and the fastest response time (1.7 ms). We also present a qualitative model to describe the device operation based on the combination of photoconductive and photogating effects. These results demonstrate the feasibility of achieving high performance in multilayer MoSe_2_ phototransistors, suggesting the possibility of further enhancement in the performance of MoSe_2_ phototransistors with proper device engineering.

## Introduction

There is a great interest in transition metal dichalcogenides (TMDs), which are composed of vertically stacked layers held together by van der Waals interactions, because of their interesting electronic, optical, and chemical properties^1,2^. Unlike graphene, the existence of bandgaps in TMDs^3,4^ such as MoS_2_ or MoSe_2_ offers an attractive possibility of using these layered materials in various device applications. Field-effect transistors (FETs) based on single or multilayer MoS_2_ exhibit outstanding performance metrics, including high on/off-current ratio (~10^7^), high mobility (~100 cm^2^V^−1^s^−1^) and low subthreshold swing (~70 mV decade^−1^)^5,6^. As the band structure of TMDs depends on their physical thickness^3,4^, FETs based on TMDs are especially promising for optoelectronic devices such as phototransistors. As the optoelectronic properties of early MoS_2_ phototransistors improved^7–10^, high responsivity (~10^5^ AW^−1^) and fast response time (~1 ms) were obtained in MoS_2_ phototransistors with device engineering such as HfO_2_ encapsulation or ferroelectric gate dielectrics^11–13^.

While MoS_2_ has been the most extensively investigated TMD for device applications, the higher optical absorbance of MoSe_2_^14^ suggests that MoSe_2_ could be more suitable than MoS_2_ for the application of phototransistors. However, little attention has been given to the optoelectronic properties of MoSe_2_ phototransistors, which has been less impressive than those of MoS_2_ phototransistors (responsivity: 0.01–238 AW^−1^, response time: 5–400 ms)^13,15–19^. Therefore, in this study, we explore the optoelectronic properties of MoSe_2_ phototransistors fabricated with mechanically-exfoliated multilayer flakes on SiO_2_/Si substrates. Our best-performance MoSe_2_ phototransistor in a simple bottom-gate thin-film transistor configuration exhibits high responsivity (~1.4 × 10^5^ AW^−1^) and fast response time (~1.7 ms) under 650-nm-laser surpassing previously reported MoSe_2_ phototransistors. We also investigate the dependence of photocurrent on gate voltage and optical power density to describe the device operation based on photoconductive and photogating effects. These results demonstrate the feasibility of achieving high performance in MoSe_2_ phototransistors without complicated device structures, suggesting that the performance of MoSe_2_ phototransistors could be further enhanced by the combination of optimized device architecture and processing.

## Results and Discussion

**Figure 1:**
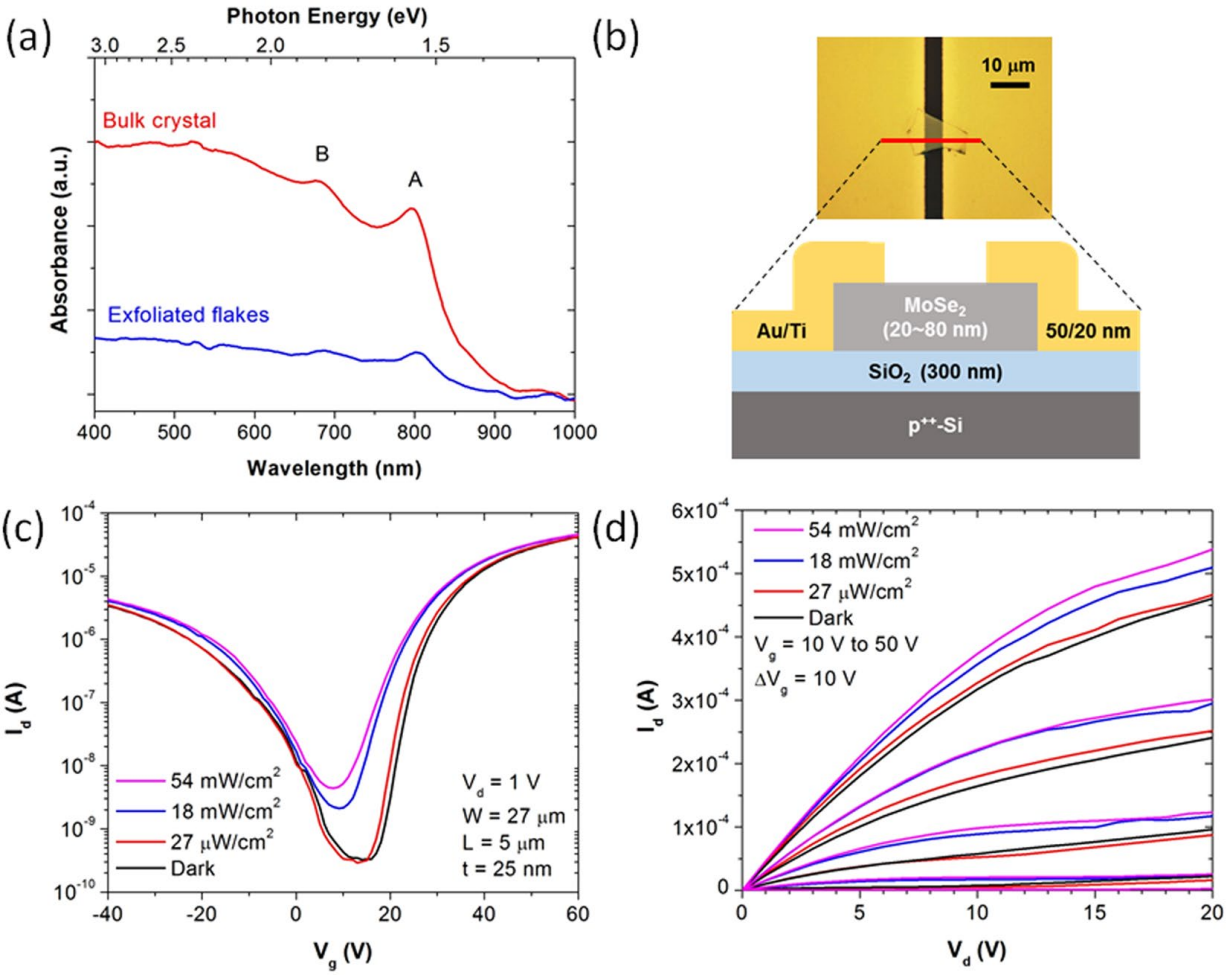
(**a**) Absorbance spectra of MoSe_2_ crystals and mechanically exfoliated flakes on sapphire with two excitonic peaks A and B, (**b**) optical microscopy image and schematic cross-section of an MoSe_2_ phototransistor along the red line, (**c**) *I*_*d*_ − *V*_*g*_ and (**d**) *I*_*d*_ − *V*_*d*_ characteristics of an MoSe_2_ phototransistor with different optical power of incident light.

Before fabricating MoSe_2_ transistors, we first measure the optical absorbance of MoSe_2_ crystals and mechanically exfoliated flakes on sapphire substrates across visible and near-infrared spectral ranges (Fig. 1(a)). The MoSe_2_ crystal is thicker than 100 μm and the thickness of exfoliated MoSe_2_ flakes are in the range of 20–80 nm. Both samples show two excitonic absorbance peaks A and B at 1.55 eV and 1.82 eV, respectively, which is consistent with literature^20^. Next, multilayer MoSe_2_ transistors are fabricated on SiO_2_/Si substrates. Figure 1(b) shows the optical microscopy image of a completed MoSe_2_ transistor along with its schematic cross-section. The measured transfer curve of an MoSe_2_ transistor in Fig. 1(c) shows asymmetric ambipolar behavior with strong *n*-type characteristic (MoSe_2_ thickness (t) = 25 nm). For electron transport without light, the MoSe_2_ transistor exhibits on/off-current ratio (*I*_*on*_*/I*_*off*_) of 10^5^ and field-effect mobility (*μ*_*FE*_) of 50.6 cm^2^V^−1^s^−1^ extracted from *μ*_*FE*_ = *L(dI*_*d*_*/dV*_*g*_)*/(WC*_*ox*_*V*_*d*_*)*, where *L* is the channel length (5 μm), *I*_*d*_ is drain current, *V*_*g*_ is gate voltage, *W* is the channel width (27 μm), *C*_*ox*_ is the oxide capacitance, and *V*_*d*_ is the drain voltage (1 V). For hole transport without light, *I*_*on*_*/I*_*off*_ of 10^4^ and *μ*_*FE*_ of 2.8 cm^2^V^−1^s^−1^ are obtained. The *n*-type-dominant ambipolar behavior of MoSe_2_ transistors with Ti/Au electrodes was also observed in literature^21,22^. The output curves in Fig. 1(d) show linear region at low *V*_*d*_ suggesting decent contact properties. The transfer and output characteristics of an MoSe_2_ transistor in Fig. 1(c,d) show the increase of *I*_*d*_ with the power density of incident light.

The photocurrent (*I*_*ph*_) of phototransistors based on transition metal dichalcogenides such as MoSe_2_ is known to be dominated by photoconductive effect and photogating effect^23^. In photoconductive effect, photogenerated excess carriers increase conductivity resulting in increased current. The photocurrent component flowing between two electrodes by photoconductive effect is given by^24^ ${I}_{ph}=({\rm{\Delta }}\sigma )EWD=({\rm{\Delta }}n)q\mu EWD=q(\eta {P}_{in}/h\nu )(\mu \tau E/L)$, where *Δσ*, *E*, *D*, *Δn*, *q*, *μ*, *η*, *P*_*in*_, *h*, *ν*, and *τ* are change in conductivity, electric field, depth of absorption region, change in carrier concentration, unit charge, carrier mobility, quantum efficiency, incident optical power, Planck constant, frequency of incident light, and carrier lifetime, respectively. The photoconductive component of *I*_*ph*_ is proportional to areal power density of incident light *P*_*in*_ and weakly depends on *V*_*g*_^23,24^. In photogating effect, one type of photogenerated carriers (electrons or holes) is trapped in localized states and the other type of carriers flows in the channel unrecombined. As this is equivalent to doping by the other type of carriers, photogating effect accompanies a shift of threshold voltage (*V*_*th*_)^25^. As *V*_*th*_ shifts, the drain current changes from *I*_*d*_ to *I*_*d*_ + *ΔI*_*d*_ and it follows that^26^ ${I}_{ph}={I}_{d}({V}_{g}-{V}_{th}+{\rm{\Delta }}{V}_{th})-{I}_{d}({V}_{g}-{V}_{th})\approx {g}_{m}{\rm{\Delta }}{V}_{th}={g}_{m}(kT/q)\mathrm{ln}(1+\eta q\lambda {P}_{in}/{I}_{dark}hc)\,$, where *g*_*m*_, *k, T*, λ, *I*_*dark*_, and *c* are transconductance, Boltzmann constant, temperature, wavelength of incident light, dark current, speed of light, respectively. Thus, the photogating component of *I*_*ph*_ shows logarithmic dependence on *P*_*in*_ and is roughly proportional to transconductance (*g*_*m*_)^23,26^.

**Figure 2:**
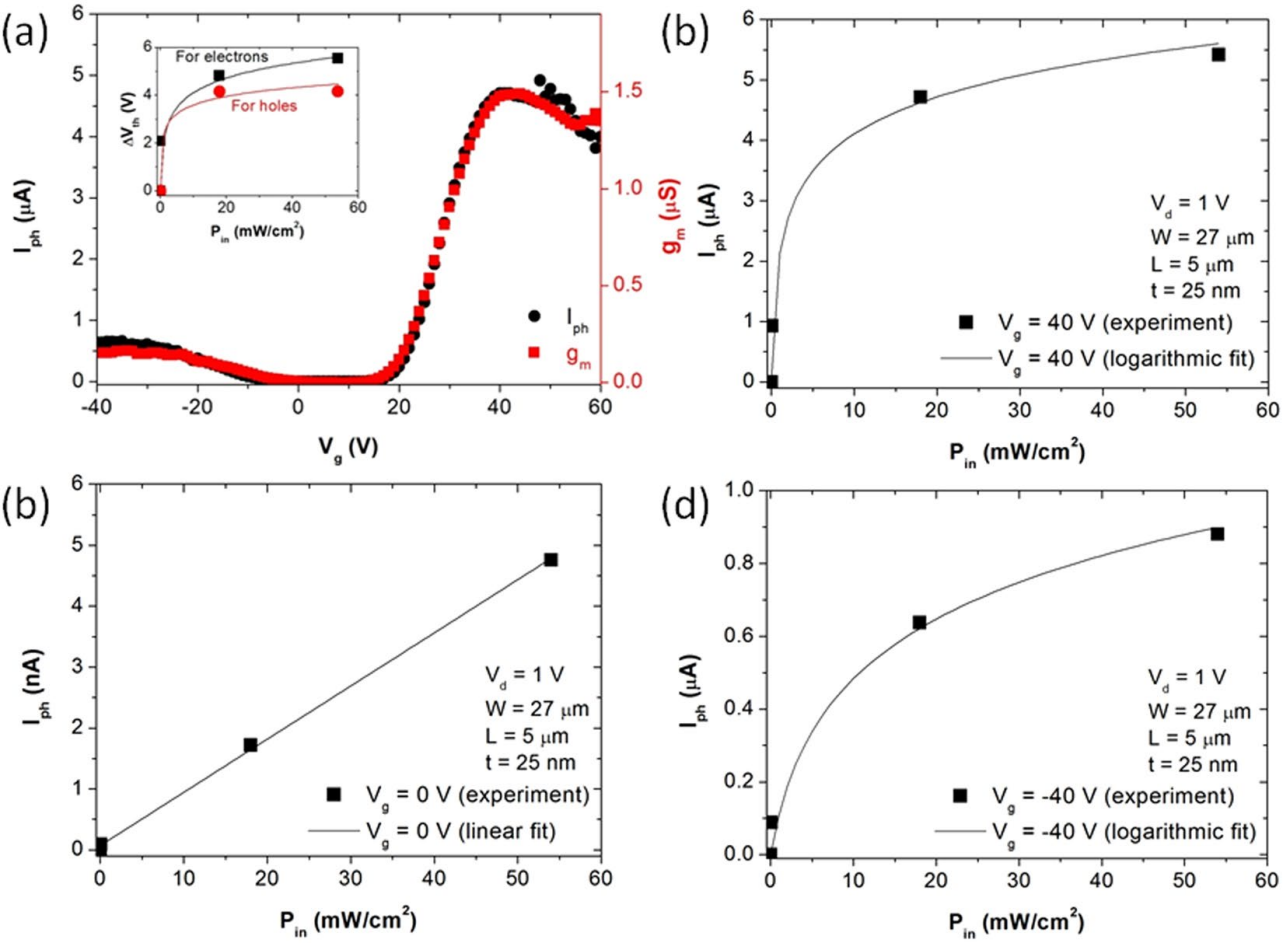
(**a**) *I*_*ph*_ and *g*_*m*_ as a function of *V*_*g*_; inset shows *ΔV*_*th*_ for electrons and holes as a function of *P*_*in*_ (solid lines: logarithmic fit); *I*_*ph*_ as a function of *P*_*in*_ (**b**) at *V*_*g*_ = 40 V, (**c**) at *V*_*g*_ = 0 V, and (**d**) at *V*_*g*_ = −40 V.

Figure 2(a) shows *I*_*ph*_ and *g*_*m*_ as a function of *V*_*g*_ for the same device in Fig. 1(c). The calculation of *I*_*ph*_ (*I*_*ph*_ = *I*_*light*_ – *I*_*dark*_, where *I*_*light*_ is *I*_*d*_ in a detector with light), and *g*_*m*_ (*g*_*m*_ = *dI*_*d*_*/dV*_*g*_) is based on the data in Fig. 1(c) at *P*_*in*_ = 18 mWcm^−2^. The similarity between *I*_*ph*_ and *g*_*m*_ suggests that photogating effect dominates the photoresponse of MoSe_2_ transistors. In the inset of Fig. 2(a), the change in *V*_*th*_ (*ΔV*_*th*_) for electrons and holes is shown as a function of *P*_*in*_. The increasing change of *V*_*th*_ with increasing *P*_*in*_ also suggests the domiant role of photogating effect in our MoSe_2_ phototransistors. However, the dependence of *I*_*ph*_ on *V*_*g*_ in Fig. 2(b) through (d) suggests that each effect dominates *I*_*ph*_ at different range of *V*_g_. Figure 2(b) through (d) show *I*_*ph*_ of our MoSe_2_ phototransistor in an on-state for electrons (at *V*_*g*_ = 40 V), an off-state (at *V*_*g*_ = 0 V), and an on-state for holes (at *V*_*g*_ = −40 V) as a function of *P*_*in*_ in sequence. *I*_*ph*_ is calculated based on the data in Fig. 1(c). The *I*_*ph*_ in an on-state for electrons (at *V*_*g*_ = 40 V) and for holes (at *V*_*g*_ = −40 V) shows logarithmic dependence on *P*_*in*_ suggesting the dominant role of photogating effect. However, the linear dependence of *I*_*ph*_ in an off-state suggests the dominant role of photoconductive effect. Such a distinct dependence of *I*_*ph*_ on *V*_*g*_ regime was also observed in phototransistors based on MoS_2_^10^, MoTe_2_^27^, compound semiconductors^26^, and organic semiconductors^28^.

**Figure 3:**
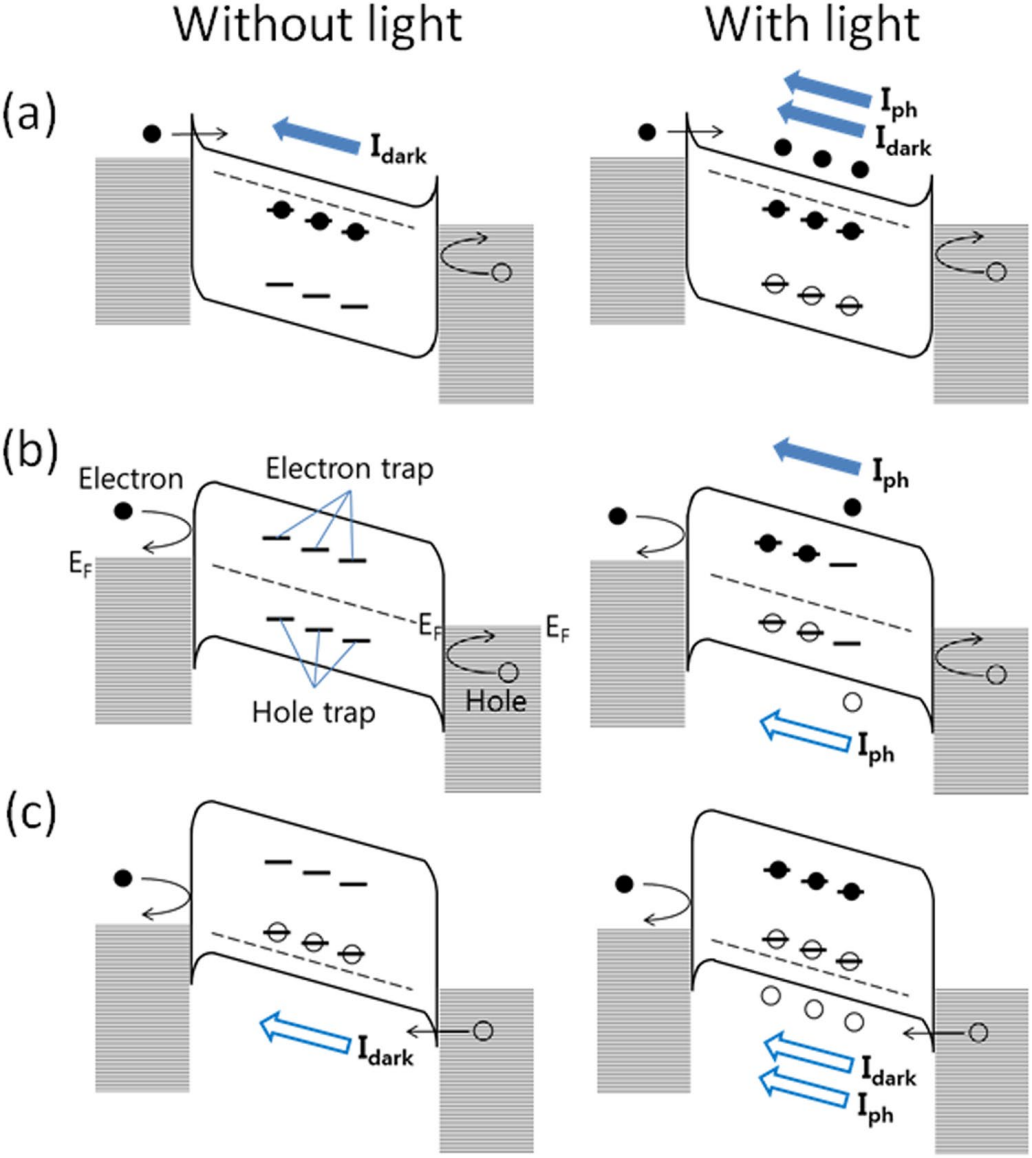
Schematic energy band diagrams of MoSe_2_ phototransistors with and without light (**a**) at *V*_*g*_ = 40 V, (**b**) at *V*_*g*_ = 0 V, and (**c**) at *V*_*g*_ = −40 V under an applied bias (*V*_*d*_).

The observed dependence of *I*_*ph*_ on *V*_*g*_ can be understood by the simplified energy band diagrams of an MoSe_2_ phototransistor under a bias (*V*_*d*_) at different *V*_*g*_ in Fig. 3. For mechanically exfoliated MoS_2_ flakes and chemical vapor deposited MoS_2_ films, the existence of trap state was reported in literature^13,29,30^ as a result of structural defects at the surface and inside MoS_2_. Similarly, we assume that electron traps and hole traps exist in the energy bandgap of MoSe_2_ by structural defects at the surface and inside MoSe_2_. In Fig. 3(a) (at *V*_*g*_ = 40 V), the Fermi level (*E*_*F*_) is located close to the conduction band edge and the majority of electron traps are filled. Without light, *I*_*dark*_ flows by the thermionic emission or tunneling of electrons. With light, the photogenerated holes fill hole traps and additional current *I*_*ph*_ flows by the unrecombined photogenerated electrons. In Fig. 3(b) (at *V*_*g*_ = 0 V), *E*_*F*_ moves toward midgap and the majority of electron traps and hole traps become unfilled. Without light, *I*_*dark*_ is negligible as the high barrier height at the contact allows negligible injection of electrons and holes. With light, *I*_*ph*_ is less than that in Fig. 3(a) as the photogenerated electrons and holes recombine or fill the trap states. In Fig. 3(c) (at *V*_*g*_ = −40 V), *E*_*F*_ is close to the valence band edge and the majority of hole traps are filled. Without light, *I*_*dark*_ flows by the thermionic emission or tunneling of holes. With light, the photogenerated electrons fill electron traps and additional current *I*_*ph*_ flows by the unrecombined photogenerated holes.

**Figure 4:**
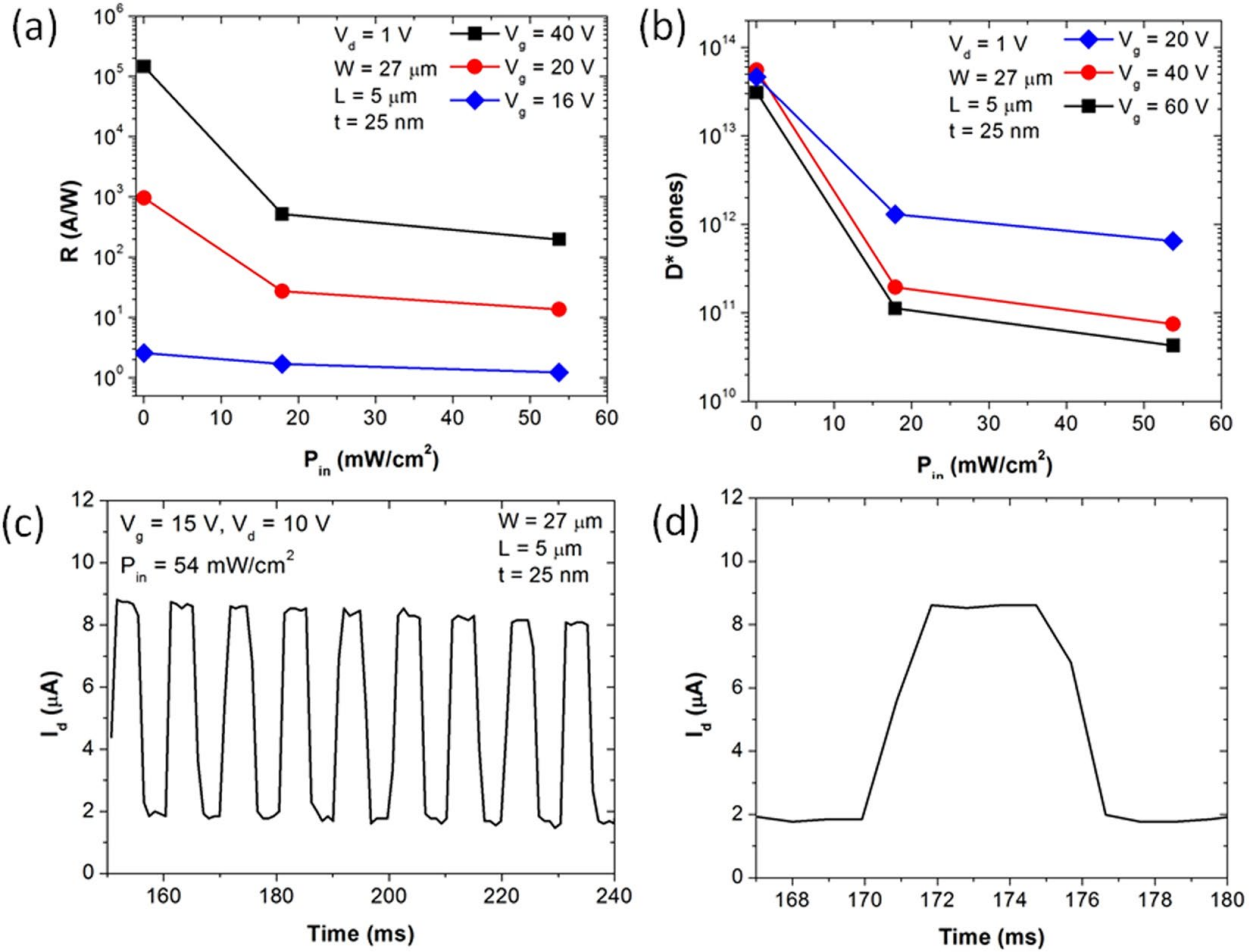
(**a**) *R* and (**b**) D* as a function of *P*_*in*_ at different *V*_*g*_; (**c**) *I*_*d*_ as a function of time and (**d**) zoomed-in region in (**c**).

The performance of an MoSe_2_ transistor as a photodetector can be evaluated by responsivity (a measure of the electrical response to light) and specific detectivity (a measure of detector sensitivity)^31^. Responsivity (*R*) is given by *R* = (*I*_*light*_ − *I*_*dark*_)/(*P*_*in*_*A*), where *A* is the area of the detector. Under the assumption that shot noise from *I*_*dark*_ is the major contributor to the total noise, specific detectivity (D*) is given by^32^ D* = *RA*^1/2^*/*(2*qI*_*dark*_)^1/2^. Figure 4(a,b) show the calculated *R* and D* of the MoSe_2_ phototransistor at different *P*_*in*_ and *V*_*g*_. Maximum *R* of 1.4 × 10^5^ AW^−1^ and D* of 5.5 × 10^13^ jones are obtained at *P*_*in*_ = 27 μWcm^−2^ and *V*_*g*_ = 40 V. These are the highest values of *R* and D* among MoSe_2_ phototransistors reported in literature so far (*R* = 0.01–238 AW^−1^ and D* = 1.0 × 10^11^–7.6 × 10^11^ jones at *P*_*in*_ = 10–100 mWcm^−2^)^13,15–19^. As *R* and D* increase with decreasing *P*_*in*_, the enhancement of *R* and D* in this work may be due to the low *P*_*in*_ compared to that in literature. However, even at comparable *P*_*in*_ in the range of 18–54 mWcm^−2^, the maximum *R* and D* in this work (*R* = 519 AW^−1^ and D* = 1.3 × 10^12^ jones) are about twice as high as those in literature. In Fig. 4(a,b), the overall dependence of *R* and D* on *P*_*in*_ and *V*_*g*_ is consistent with literature^13^. *R* increases as *P*_*in*_ decreases or *V*_*g*_ increases, while D* increases as *P*_*in*_ or *V*_g_ decreases. As *P*_*in*_ increases, more holes fill shallow trap states where lifetime is short. This results in faster recombination hence *R* decreases. When *P*_*in*_ increases, D* also decreases as *R* decreases and *I*_*dark*_ remians unchanged. When *V*_*g*_ increases, electrical doping at higher *V*_*g*_ reduces contact resistance resulting in higher photocurrent and *R*. However, as *V*_*g*_ increases, *I*_*dark*_ also increases, which degrades D*.

It needs to be mentioned that our MoSe_2_ transistors show wide device-to-device variation of *μ*_*FE*_, *R*, and D* (Table S1 in Supplementary Information). Such wide device-to-device variation is commonly observed in the transistors based on transition metal dichalcogenides such as MoSe_2_ presumably because of the variation of intrinsic defects in crystals^33^. While it is very difficult to pinpoint the origin of high performance in the best device, the correlation between *R* and *μ*_*FE*_ in this work (Fig. S1 in Supplementary Information) suggests that the enhanced optoelectronic properties may be related to the enhanced electrical performance of our MoSe_2_ device. It is also supported by the fact that our MoSe_2_ device shows the highest mobility among MoSe_2_ transistors in Table 1.

**Table 1:** Performance of MoSe_2_ phototransistors measured at *P*_*in*_ = 10–100 mWcm^−2^.

Type of MoSe_2_	*μ*_*FΕ*_ (cm^2^v^−1^s^−1^)	*R* (AW^−1^)	D* (jones)	Response time (ms)	Reference
Multilayer flake (Exfoliation)	50.6	519.2	1.3 × 10^12^	1.7 (rise) 2.2 (fall)	This work
Few layer flake (Exfoliation)	19.7	97.1	—	15 (rise) 30 (fall)	15
Single layer film (CVD)^i^	—	0.013	—	60 (rise) 60 (fall)	16
Multilayer flake (CVD)^i^	10.1	93.7	—	400 (rise) 200 (fall)	17
Multilayer flake (Exfoliation)	5.9 or 16^ii^	0.1 or 16^ii^	1.0 × 10^11ii^	5 (fall)^ii^	13
Few layer flake (Exfoliation)	1.8	0.026	—	20 (rise) 20 (fall)	18
Few layer flake (Exfoliation)	5.1	238	7.6 × 10^11^	—	19

^i^Chemical vapor deposition.

^ii^With HfO_2_ encapsulation.

We also note the negligible correlation between the optoelectronic properties of MoSe_2_ devices and MoSe_2_ thickness. This may seem counterintuitive because the width of energy bandgap changes for thin MoS_2_ crystals (< ~4 nm in thickness)^4^ and light absorption depends on MoSe_2_ thickness. Yet, because the thickness of our MoSe_2_ flakes ranges from 20 nm to 80 nm, we expect negligible differences in energy bandgap in our MoSe_2_ devices. On the other hand, we expect higher responsivity for devices with thicker MoSe_2_ as more light is absorbed in thicker MoSe_2_. However, the responsivity shows negligible correlation with thickness of MoSe_2_ flakes in this investigation (Fig. S2 in Supplementary Information). This may be due to the variation of intrinsic materials quality overshadowing the effect of thickness. The mobility and detectivity in Fig. S2 also show negligible correlation with the thickness of MoSe_2_ flakes, supporting this argument.

To explore the response time of our MoSe_2_ phototransistors, we measure the time-resolved photoresponse of our MoSe_2_ phototransistors for multiple illumination cycles. Figure 4(c) shows the result for the same device in Fig. 1(c). The incident laser with a power density of 54 mWcm^−2^ is modulated with a square wave at 100 Hz at *V*_*g*_ = 15 V and *V*_*d*_ = 10 V. The nearly identical response for multiple cycles suggests the overall rebustness and reproducibility of our MoSe_2_ phototransistors. From a zoomed-in region in Fig. 4(d), we obtain rise time of 1.7 ms and fall time of 2.2 ms. (Rise time is calculated as the time taken by current to increase from 10% to 90% of the maximum current. Fall time is calculated as the time taken by current to decrease from 90% to 10% of the maximum current.) This is the fastest response time of MoSe_2_ phototransistors ever reported in literature, which ranges from 5 ms to 400 ms^13,15–19^. It is intriguing that our MoSe_2_ phototransistors exhibit high responsivity and fast response time. Because the long lifetime of carriers in photogating effect suggests slow response to light, the fast response time in our MoSe_2_ device may be related to the characteristics of trap states. One possibility is that trap states in our MoSe_2_ device may have shorter lifetime and higher density than those in literature. Then, while the shorter lifetime of trap states could provide fast response, the higher density of trap states could provide higher doping enhancing responsivity. However, we may only speculate at this stage and further investigation is needed on the characteristics of trap states including the distribution of trap energy, trap density, trap lifetime, and carrier capture probability.

Table 1 compares *μ*_*FE*_, *R*, D*, and response time of MoSe_2_ phototransistors in literature. Because the measurement conditions, such as *V*_*g*_, *V*_*d*_, *P*_*in*_, and excitation energy, can influence the device performance, comparable measurement conditions with those in literature are used in this work. Our MoSe_2_ phototransistors exhibit the best performance in terms of *μ*_*FE*_, *R*, D*, and response time, demonstrating the feasibility of achieving high responsivity and fast response time in multilayer MoSe_2_ phototransistors. Future work combining controlled growth of materials with optimized device architecture and processing will further enhance the performance of MoSe_2_ phototransistors.

## Conclusions

We report high-responsivity multilayer MoSe_2_ phototransistors with fast response time fabricated with mechanically-exfoliated MoSe_2_ flakes on SiO_2_/Si substrates. Our MoSe_2_ phototransistors exhibit asymmetric ambipolar behavior with strong *n*-type characteristic. Without light, high on/off-current ratio of 10^5^ and field-effect mobility of 50.6 cm^2^V^−1^s^−1^ are obtained for electrons. Under 650-nm-laser, our MoSe_2_ phototransistor exhibits the best performance among MoSe_2_ phototransistors in literature including high responsivity (1.4 × 10^5^ AW^−1^), high specific detectivity (5.5 × 10^13^ jones), fast rise time (1.7 ms) and fast fall time (2.2 ms). The dependence of photocurrent on gate voltage and optical power density suggest that photocurrent is dominated by photogating effect in on-state and by photoconductive effect in off-state. These results demonstrate the feasibility of achieving high-performance multilayer MoSe_2_ phototransistors, providing potentially important implications on using MoSe_2_ phototransistors for a variety of applications including touch sensor panels, image sensors, solar cells, and communication devices.

## Methods

### Device fabrication

Multilayer MoSe_2_ flakes were obtained by gold-mediated mechanical exfoliation^34^ from bulk MoSe_2_ crystals (2D Semiconductors) and transferred to highly doped p-type Si wafer with thermally grown SiO_2_ (300 nm). The thickness of MoSe_2_ flakes measured by atomic force microscope (AFM, Park Systems XE-100) existed between 20 nm and 80 nm. To form source and drain electrodes (100 μm × 100 μm) on top of MoSe_2_ flakes, Ti (20 nm) and Au (50 nm) deposited by electron-beam evaporation were patterned using photolithography and etching. The device was then annealed at 200 °C in a vacuum tube furnace for 2 hours (100 sccm Ar and 10 sccm H_2_) to remove resist residue and to decrease contact resistance.

### Device characterization

Optical absorbance of MoSe_2_ was measured by UV-visible spectroscopy (Perkin-Elmer Lambda 35). Electrical characterizations were carried out with current-voltage (*I*-*V*) measurements (Agilent 4155 C Semiconductor Parameter Analyzer) at room temperature. The photoresponse of MoSe_2_ phototransistors was measured with a 650-nm-laser (beam size of 3 mm) at different power densities (0.027, 18 and 54 mW cm^−2^). Dynamic on/off switching was conducted using a function generator (Tekronix AFG310).

## Electronic supplementary material

Supplementary Information
