# Supplementary material for: High-Responsivity Multilayer MoSe2 Phototransistors with Fast Response Time
Source: Sci Rep. 2018 Aug 1;8:11545. doi: 10.1038/s41598-018-29942-1 (PMC6070481; doi:10.1038/s41598-018-29942-1)
Supplement: Supplementary file 1 — Supplementary Information [file 41598_2018_29942_MOESM1_ESM.docx]

**Supplementary Information**

High-Responsivity Multilayer MoSe_2_ Phototransistors with Fast Response Time

Hyejoo Lee^1^, Jongtae Ahn^2^, Seongil Im^2^, Jiyoung Kim^3^, and Woong Choi^[[1]](#footnote-1)*^

^1^ School of Materials Science & Engineering, Kookmin University, Seoul 02707, South Korea

^2^ Institute of Physics and Applied Physics, Yonsei University, Seoul 03722, South Korea

^3^ Department of Materials Science & Engineering, University of Texas at Dallas, Richardson, Texas 75080, USA

1. Device-to-device variation of device performance

2. Correlation between responsivity and mobility

3. Effect of MoSe_2_ thickness on responsivity, mobility, and detectivity

1. Device-to-device variation of device performance

Table S1. Field-effect mobility, responsivity, and detectivity of each device along with MoSe_2_ thickness.

| Sample | Field-effect mobility (cm^2^/Vs) | Responsivity (A/W) | Detectivity (jones) | MoSe_2_ thickness (nm) |
| --- | --- | --- | --- | --- |
| 1 | 53.30 | 7.56×10^3^ | 8.76×10^12^ | 55 |
| 2 | 50.60 | 1.47×10^5^ | 4.81×10^13^ | 25 |
| 3 | 14.30 | 4.07×10^4^ | 1.01×10^13^ | 50 |
| 4 | 20.60 | 1.68×10^4^ | 1.15×10^13^ | 70 |
| 5 | 30.20 | 3.09×10^3^ | 5.16×10^12^ | 55 |
| 6 | 9.41 | 2.24×10^2^ | 1.45×10^12^ | 43 |
| 7 | 45.68 | 3.50×10^3^ | 3.94×10^12^ | 70 |
| 8 | 0.93 | 7.04×10^2^ | 4.33×10^12^ | 68 |
| 9 | 11.12 | 3.50×10^3^ | 4.59×10^12^ | 15 |
| 10 | 13.65 | 4.99×10^4^ | 2.50×10^13^ | 63 |
| 11 | 2.99 | 1.46×10^4^ | 6.76×10^12^ | 15 |
| 12 | 0.53 | 4.12×10^2^ | 5.32×10^12^ | 40 |
| 13 | 2.84 | 4.53×10^3^ | 1.07×10^13^ | 33 |
| 14 | 50.90 | 7.82×10^3^ | 1.54×10^13^ | 31 |
| 15 | 25.10 | 1.57×10^3^ | 4.65×10^12^ | 20 |
| 16 | 23.50 | 2.57×10^4^ | 3.83×10^13^ | 30 |
| 17 | 43.40 | 5.28×10^4^ | 1.63×10^13^ | 42 |
| 18 | 15.80 | 7.87×10^3^ | 1.84×10^13^ | 19 |
| Average | 23.05 | 2.15×10^4^ | 1.33×10^13^ | 41.33 |
| Standard deviation | 18.46 | 3.55×10^4^ | 1.26×10^13^ | 19.08 |

2. Correlation between responsivity and mobility


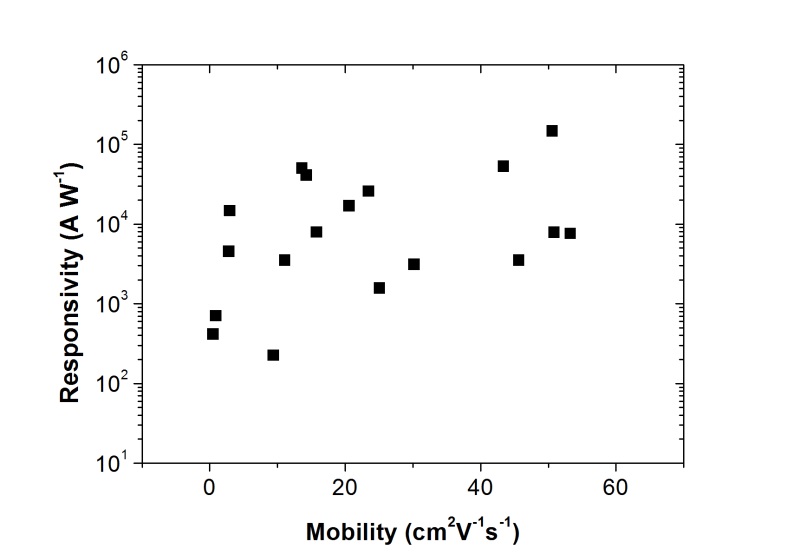


Figure S1. Responsivity and mobility of MoSe_2_ devices in this work

3. Effect of MoSe_2_ thickness on responsivity, mobility, and detectivity


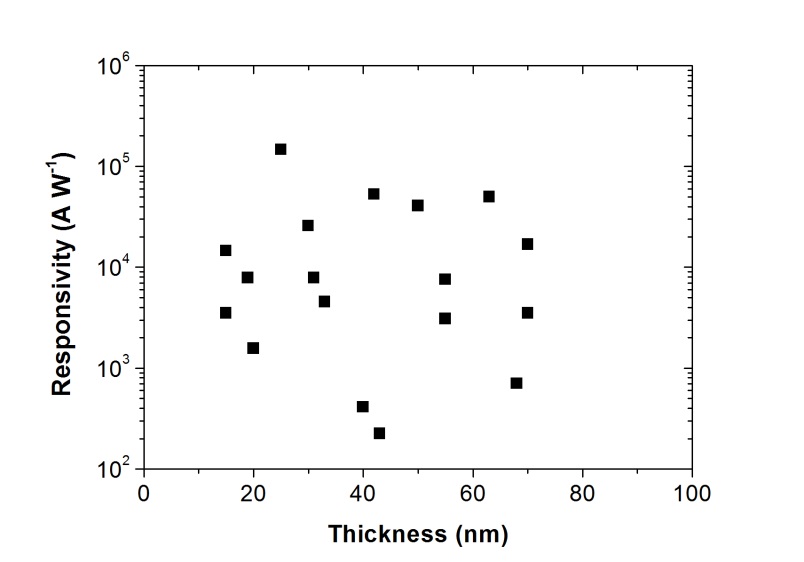


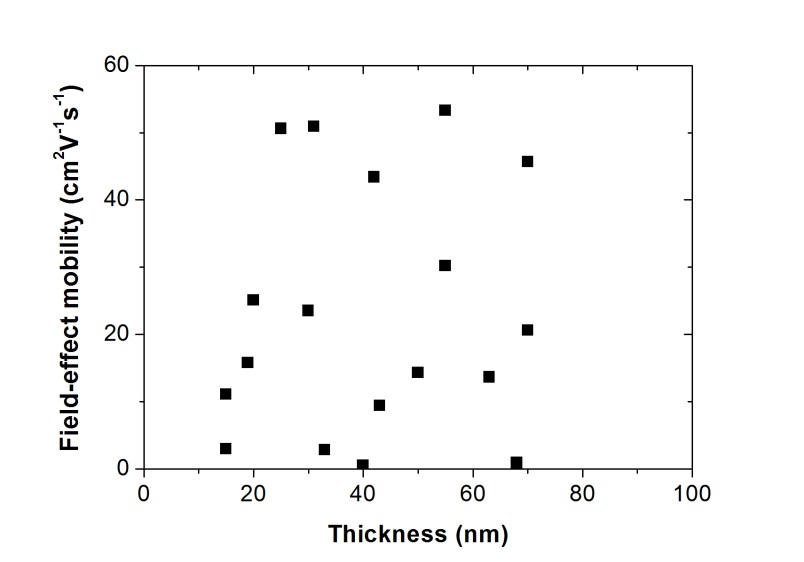


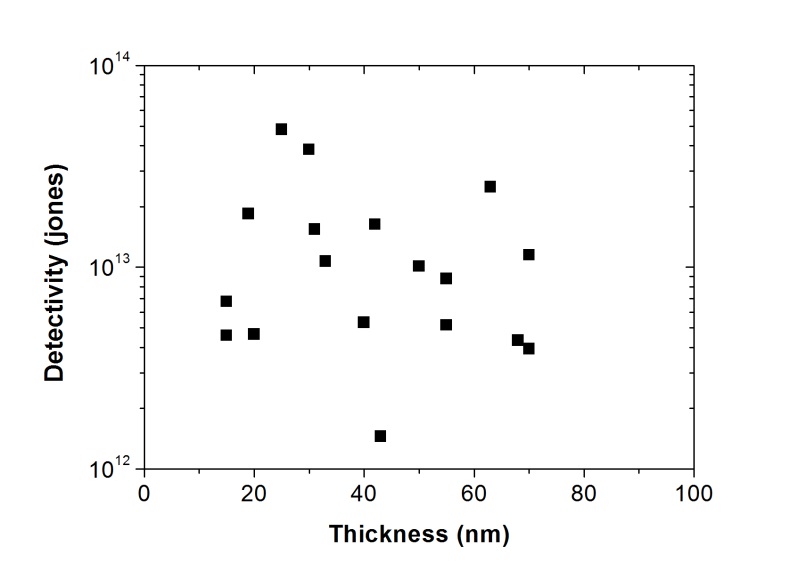


Figure S2. Responsivity, field-effect mobility, and detectivity of MoSe_2_ phototransistors as a function of its thickness.

1. * E-mail: woongchoi@kookmin.ac.kr [↑](#footnote-ref-1)
